# The relationship between minor coronal asymmetry of the spine and measures of spinal sagittal shape in adolescents without visible scoliosis

**DOI:** 10.1038/s41598-023-31237-z

**Published:** 2023-03-15

**Authors:** Adrian Gardner, Fiona Berryman, Paul Pynsent

**Affiliations:** 1 University of Birmingham, Birmingham, UK; 2 The Royal Orthopaedic Hospital NHS Foundation Trust, Birmingham, UK

**Keywords:** Risk factors, Experimental models of disease, Anatomy, Musculoskeletal system

## Abstract

The purpose of this work is to identify what features of overall spinal sagittal shape are associated with coronal asymmetry in those without scoliosis. Using a longitudinal analysis of Integrated Shape Imaging System 2 (ISIS2) surface topography images of those without scoliosis, measures of coronal asymmetry, along with measures of spinal sagittal shape (kyphosis, lordosis and sagittal imbalance, which is a measure of the position of the top of the thoracic spine relative to the sacrum) were analysed using linear mixed effect models (LMEM), which is a method of analysing the components of a complex model (such as that describing overall spinal shape), to ascertain the relative relationships between the parameters. Data was also analysed when subdivided for the anatomical level of coronal asymmetry (thoracic or thoracolumbar/lumbar pattern). There were 784 measures from 196 children. Kyphosis had little effect on coronal asymmetry for males and females, lordosis increased with coronal asymmetry in females only and sagittal imbalance increased with coronal asymmetry in males only. The results of the LMEM modelling were that the parameters related to coronal asymmetry were lordosis and sagittal imbalance. In thoracic coronal asymmetry, whilst lordosis was predominant, kyphosis played more of a role. In thoracolumbar/lumbar coronal asymmetry, lordosis and sagittal imbalance were the larger coefficients. Coronal asymmetry of the spine in those without scoliosis is related to features of spinal sagittal shape, particularly lordosis and sagittal imbalance. This knowledge adds to the understanding of the aetiology of adolescent idiopathic scoliosis.

## Introduction

Minor coronal asymmetry of the spine, defined as the spine not being straight in the coronal plane, is common in adolescents without scoliosis^1^. An increasing coronal asymmetry becomes pathological, and is defined as scoliosis by a Cobb angle of 10° or more in the coronal plane with associated intervertebral rotation^2^. Adolescent idiopathic scoliosis (AIS) is a three-dimensional (3D) helical deformity of the spine^2^, known to be associated with a change in the sagittal shape of the spine^3,4^. Demonstrating radiographically how a scoliotic spine develops in a child with a straight spine is challenging as it would require multiple radiographs in a large number normal children in a longitudinal fashion and is unethical due to the cumulative risks of ionising radiation^5^. For these two reasons, it is not clear what parameters that describe the 3D shape of the spine might lead to the subsequent development of scoliosis.

Previously, Dolphens et al.^6^ published a cross-sectional study of children without scoliosis who were around their pre-pubertal growth spurt. These volunteers were examined for coronal asymmetry of the spine by palpation.The presence or absence of coronal asymmetry was analysed against measures of overall whole body sagittal shape (the sagittal shape of the whole child, measured from the gravity line from a photograph that covered from the top of the head to the soles of the feet) using logistic regression and this included the size of the thoracic kyphosis and lumbar lordosis, along with an assessment of the overall sagittal posture. Dolphens showed that coronal asymmetry was associated with whole body sagittal shape and more often seen with a ‘non-neutral’ sagittal shape, described in their paper as a ‘sway back’ or ‘leaning forwards’ posture. The authors acknowledge the cross sectional nature of the study as a possible methodological issue, highlighting the difficulties of performing longitudinal studies in normal children.

More recently, using a surface topography method, our group has observed differences in the development of both kyphosis and lordosis in a cohort of adolescents without scoliosis who were measured and analysed in a truly longitudinal fashion^7,8^. Females are more lordotic than males with lordosis increasing with age for both sexes. Kyphosis on the other hand increases in males, tailing off towards the end of adolescence, whilst in females there is a decrease in kyphosis between the ages of 11 and 13 before returning to previous levels. A hypothesis linking the development of both kyphosis and lordosis in the growing spine and an association with the development of AIS was recently advanced^8^.

This current paper has two aims. First, is to examine the relationships between small amounts of coronal asymmetry and the sagittal shape of the spine, to improve knowledge of the aetiology of AIS. Second, to re-examine the conclusions of the paper of Dolphens et al.^6^ with a longitudinal cohort. This will be done by eliminating the change in the parameters that describe the shape of the spine that occurs during the adolescent growth spurt to reveal the relationship between the parameters irrespective of age.

## Methods

All methods described were carried out in accordance with relevant UK Health Research Authority guidelines and procedures^9^. All experimental protocols were approved by the National Research Ethics Service UK (NRES committee West Midlands—South Birmingham (11/H1207/10)). Written informed consent was obtained from the parent or guardian of all subjects, with written assent given by the child themselves.

The data is the longitudinal measurement of spinal shape in a group of children and adolescents between 2011 and 2017 at a local school. The inclusion criteria were an age of between 9 and 18 years along with a willingness to take part in the research, to allow serial measures being taken every year until the child left full time education (in the year that the child become 18 years of age). The exclusion criteria were (i) a history of either scoliosis (ii) a pathology of the bony torso, (iii) previous treatment for scoliosis (bracing or surgery) or (iv) any sort of bracing of the thorax (for a pectus deformity) or surgery on the bony thorax or intra-thoracic organs. Enrolled children came every year for an ISIS2 image of their spine and torso.

The measures were taken using the Integrated Shape Imaging System 2 (ISIS2), measuring surface topography of the back and spine^10^. Measures were taken yearly for children aged between 9 and 18 years. Other aspects of this data set have been reported on previously^1,7,8^.

Measures of coronal asymmetry for all of the data (noting that the majority of the children were measured on multiple occasions) were analysed with regard to measures of coronal asymmetry and sagittal spinal shape, namely kyphosis, lordosis and sagittal imbalance. For measures of coronal asymmetry, all curves were treated as having a positive value irrespective of the side of the convexity (as preliminary analysis did not demonstrate a difference between a convexity to the left or right).

To understand the methodology of this paper, a brief explanation of the ISIS2 system is required. Following on from the work of Turner Smith et al.^11^, ISIS2^10,12^ uses a similar system of data capture through distortion of parallel light fringes caused by the shape of the body. The method of use of ISIS2 is standardised^10^. The child is stood comfortably with their body weight equally shared between both lower limbs and their arms by their sides. An abdominal bar is present in front of them to allow positional feedback and prevent sway, although this is not leant on. The ISIS2 system is different when compared to a lateral radiograph as ISIS2 gives a true representation of upright stance as the arms are not required to be placed in a fist on clavicle or similar position to allow imaging of the upper thoracic spine through the shoulder girdle^13^. Whilst the application of stickers identifies surface landmarks (VP, posterior superior iliac spines (PSIS), spinous processes), the combination of the shape of the posterior torso along with a 5th order polynomial that passes through those surface markers is adjusted via a previously validated algorithm to a 3D line that demonstrates the position of the vertebral bodies, and this is the data that this paper is based upon. Thus whilst it is correct to note that at no point did any of the participants within the study undergo a radiograph to identify the bony anatomy, the data of spinal shape that is generated from the ISIS2 system is of the spine and not of the skin surface. The point that is labelled as the marker of the sacrum is a point that is generated by the system and is at the midpoint of a line that joins both of the PSIS, as part of the spine line and not only a surface marker. It is true to note that the anatomical location of the sacral point is not the same as the anatomical point of the posterior superior corner of the S1 vertebral body (as is used in a radiograph for measures of sagittal alignment), however, as the measure of sagittal alignment from both ISIS2 and a radiograph is a linear rather than angular measure, measuring the distance between vertical lines through the sacrum and the C7 vertebral body, and as such the length of the vertical lines is not relevant to the measure.

Sagittal imbalance describes the relationship between the position of the VP and sacrum in the sagittal plane as a horizontal distance (in millimetres) between vertical lines through the VP and sacrum. When the VP is anterior to sacrum, sagittal imbalance is defined as positive. Coronal asymmetry is defined within the ISIS2 surface topography system as the angle subtended between the points of inflection of the spinal curves in the coronal projection, in a similar fashion to the Cobb angle^14^. The measures of kyphosis and lordosis are taken in the same way in the sagittal plane as in the coronal plane (in a similar fashion to the Cobb angle^14^) with kyphosis measured between the Vertebra Prominens (VP) and the point of inflection (where the kyphosis becomes lordosis) around the thoracolumbar junction between the kyphotic and the lordotic curves. Lordosis is measured between the point of inflection and the sacrum.”

The ISIS2 system has been described in the literature and validated against radiographs for a number of parameters in both scoliosis and kyphosis^10,12,15,16^. The inter and intra-observer measurement errors for ISIS2 are documented^17^.

The R Statistical Computing Platform^18^ was used for the analysis together with the ggplot2 R package^19^. The longitudinal data for each parameter was plotted to demonstrate the relationships between coronal asymmetry and either kyphosis, lordosis or sagittal imbalance. The data are shown as a series of lines representing multiple longitudinal measurements over time for each individual in a spaghetti plot. The mean (solid blue line) and 95% confidence intervals (grey funnel) of that data are demonstrated using loess regression. The statistical significance between the sagittal profile parameters, compared to the coronal asymmetry, of males and females for the data was assessed by using an ANOVA test, comparing of the model with the parameter of interest against the same model without the parameter of interest, with statistical significance pre-defined as p < 0.05 following a power analysis appropriate to the data.

To understand the relationships between the measured parameters further, a linear mixed effect model (LMEM) was created using the R lme4 package^20,21^ using both random intercepts and gradients. The model was created with the fixed effects of the measures of sagittal shape (kyphosis, lordosis and sagittal imbalance) and the random effects of multiple measures from the same individual. The magnitude of the individual fixed effects in the model were visualised using the R effects package^22^.

As there were differences noted when the data was subdivided into a thoracic or thoracolumbar/lumbar location in the paper of Dolphens^6^, the data presented here was also analysed dependant on whether the coronal asymmetry seen was a thoracic or thoracolumbar/lumbar level.

## Results

The analysed data comprised 784 measures in 196 children. As this data collection was a longitudinal study in a school over seven years, not all children were measured on every occasion. There were 164 children were measured on three or more occasions (739 longitudinal measures within the total of 784). There were 117 males and 79 females in total. The demographics along with the mean value, standard deviation and range of coronal asymmetry, kyphosis, lordosis and sagittal imbalance are seen in Table 1.

**Table 1:** Table of demographics and number of measures.

	Males	Females
Number of individuals	117 (60%)	79 (40%)
Age range (years)	9.3 to 18.0	9.2 to 16.4
Number of individuals measured on 3 or more occasions	104 (63%)	60 (37%)
Number of individual measures	484 (62%)	300 (38%)
Total number of measures from individuals measured on 3 or more occasions	466 (63%)	273 (37%)
Number of main thoracic curves	305 (64%)	172 (36%)
Number of main thoracolumbar/lumbar curves	179 (58%)	128 (42%)
Coronal asymmetry (°) as mean (standard deviation, range)	5 (3, 0 to 14)	6 (3, 0 to 15)
Kyphosis (°) as mean (standard deviation, range)	35 (8, 15 to 61)	34 (10, 4 to 57)
Lordosis (°) as mean (standard deviation, range)	28 (10, 5 to 58)	31 (9, 8 to 52)
Sagittal imbalance (mm) as mean (standard deviation, range)	30 (18, -32 to 83)	20 (19, -39 to 81)

**Figure 1:**
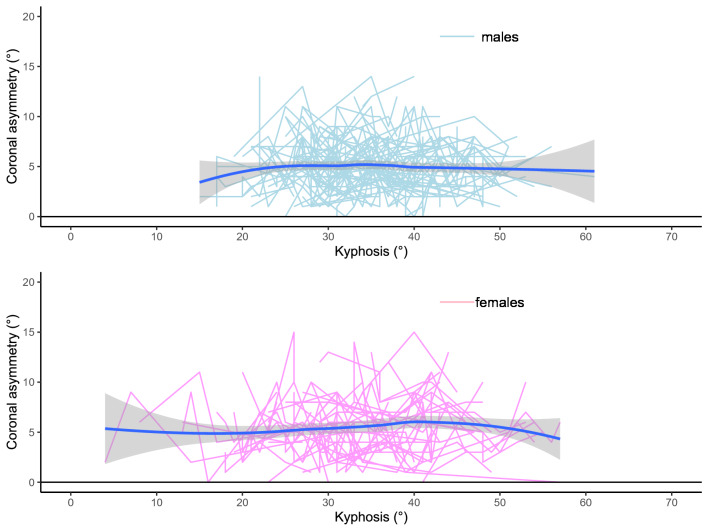
Spaghetti plot of kyphosis (°) against coronal asymmetry (°).

**Figure 2:**
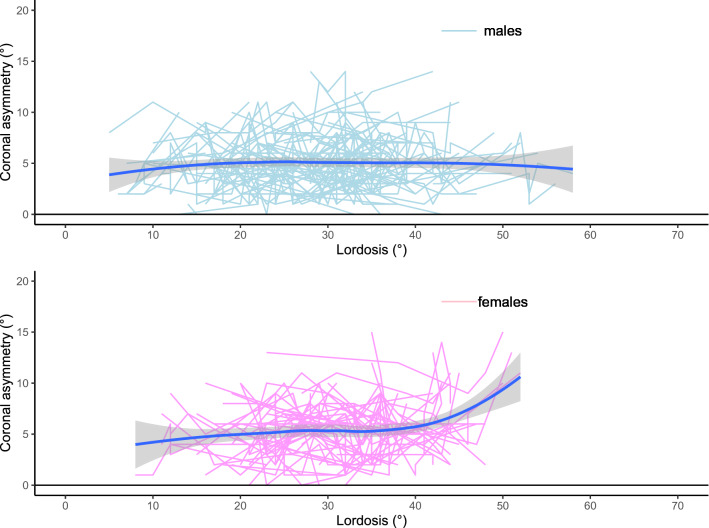
Spaghetti plot of lordosis (°) against coronal asymmetry (°).

**Figure 3:**
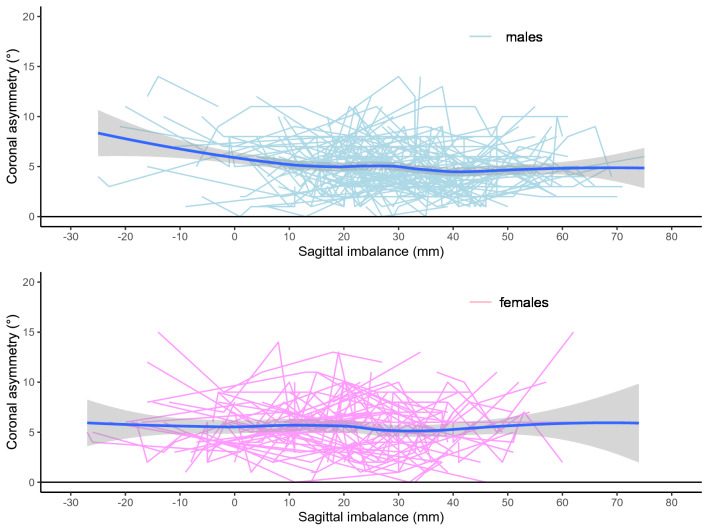
Spaghetti plot of sagittal imbalance (mm) against coronal asymmetry (°).

The spaghetti plots of kyphosis, lordosis and sagittal imbalance, plotted against coronal asymmetry, are shown in Figs. 1, 2, 3. These plots show that a change in the amount of kyphosis is not associated with a change in the amount of coronal asymmetry for either males or females. For lordosis however, there is a difference between males and females. In males, a change in lordosis does not lead to a change in coronal asymmetry but in females there is an increase in coronal asymmetry with an increase in lordosis, particularly at higher levels (> 35°). For sagittal imbalance, there is a decrease in coronal asymmetry for an increase in sagittal imbalance in males but no change in females. However, none of the three parameters demonstrated a statistical significance between males and females (p > 0.2 in all cases noting that this is analysis is underpowered due to the very small effect size between the groups).

**Figure 4:**
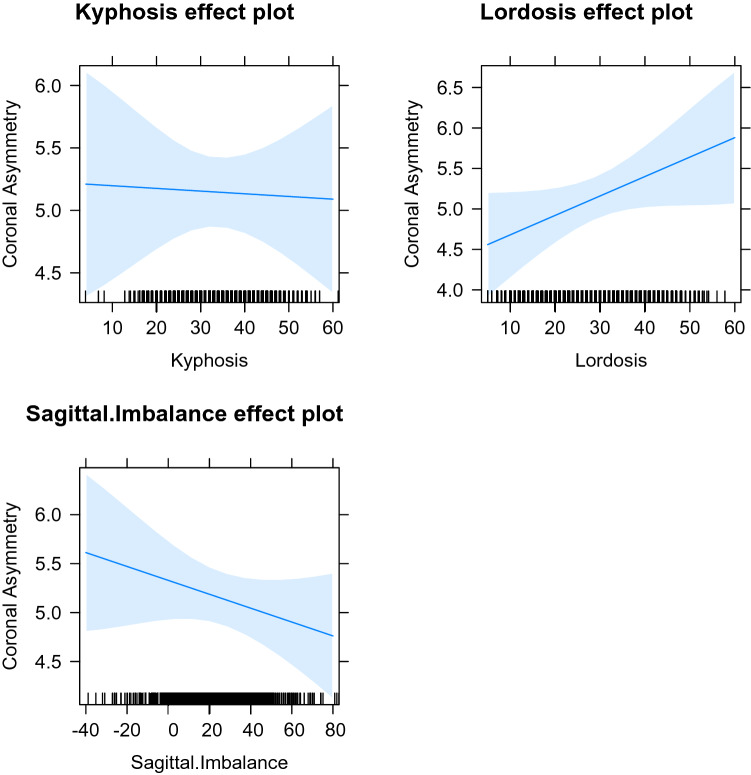
All effects model plot all data.

**Figure 5:**
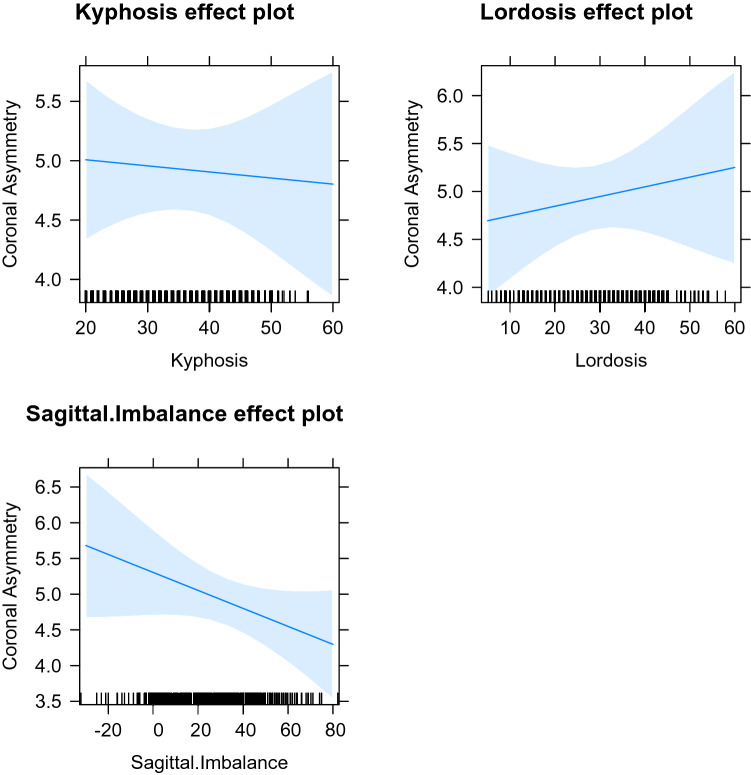
All effects model plot males.

**Figure 6:**
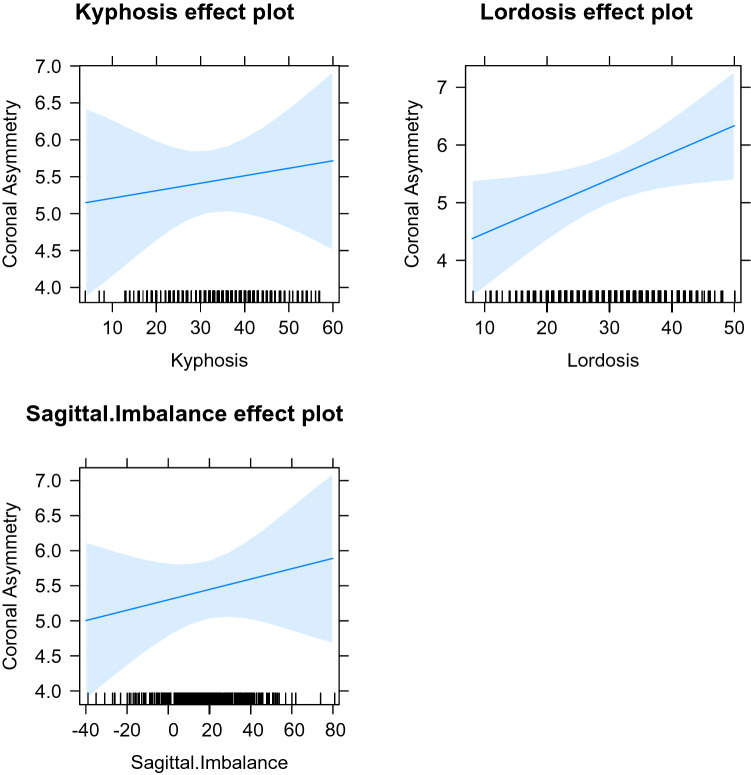
All effects model plot females.

The LMEM model was examined, combining the data from both coronal asymmetry types, using all of the data from males and females together, and then separately, for all three measures of sagittal shape (Figs. 4, 5, 6). When examined without dividing for sex, the model demonstrates that kyphosis has little effect on coronal asymmetry whereas an increase in lordosis increases coronal asymmetry. Sagittal imbalance has a negative effect on coronal asymmetry. When the LMEM model was formed with males only, again increasing lordosis leads to increasing coronal asymmetry, although not as marked. Sagittal imbalance has a slightly greater negative effect on coronal asymmetry. Kyphosis exhibits little effect on coronal asymmetry. A model composed of females only demonstrates differences to the all data, and to the males only, models. An increase in all three measures leads to an increase in coronal asymmetry, the greatest being lordosis. The coefficients are seen in Table 2.

**Table 2:** The fixed coefficients (with standard error in brackets) of the models for both types of main coronal asymmetry.

	All data	Males	Females
Kyphosis	− 0.002 (0.014)	− 0.005 (0.019)	0.01 (0.021)
Lordosis	0.024 (0.013)	0.01 (0.015)	0.047 (0.021)
Sagittal imbalance	− 0.007 (0.006)	− 0.013 (0.008)	0.007 (0.009)

When the data was subdivided for the anatomical location of the area of coronal asymmetry, the results for thoracic coronal asymmetry were different from those with thoracolumbar/lumbar coronal asymmetry (Tables 3 and 4). For thoracic (both sexes), kyphosis contributed more of a role, and sagittal imbalance less, than for all locations of coronal asymmetry combined, noting however that lordosis was the predominant parameter. The differences between males and females for thoracic coronal asymmetry is the relative difference in the contributions of kyphosis and lordosis. Lordosis is the larger coefficient especially in females, but with kyphosis being greater in males than in females. Whilst smaller in magnitude, sagittal imbalance was larger in females than in males. For thoracolumbar/lumbar coronal asymmetry with the sexes combined, lordosis and sagittal imbalance were the main contributors. When subdivided for sex, the thoracolumbar/lumbar coronal asymmetry shows lordosis being a larger coefficient followed by sagittal imbalance for males and kyphosis in females. Of note, sagittal imbalance changes from a negative to a positive coefficient when comparing females to males, or all data combined, for thoracolumbar/lumbar coronal asymmetry.

**Table 3:** The fixed coefficients (with standard error in brackets) of the models for a main thoracic coronal asymmetry pattern.

	All data	Males	Females
Kyphosis	− 0.017 (0.017)	− 0.015 (0.022)	− 0.009 (0.026)
Lordosis	0.025 (0.025)	0.018 (0.018)	0.028 (0.029)
Sagittal imbalance	− 0.001 (0.007)	− 0.001 (0.009)	− 0.005 (0.012)

**Table 4:** The fixed coefficients (with standard error in brackets) of the models for a main thoracolumbar/lumbar coronal asymmetry pattern.

	All data	Males	Females
Kyphosis	0.011 (0.022)	0.011 (0.022)	0.021 (0.031)
Lordosis	0.023 (0.019)	0.023 (0.019)	0.066 (0.028)
Sagittal imbalance	− 0.015 (0.009)	− 0.016 (0.009)	0.008 (0.013)

## Discussion

There are a number of theories that have been advanced to describe the aetiology of AIS^23–27^. It is recognised that an alteration in the relationships within the sagittal profile of the spine is key to this process^22–28^. The Utrecht group have published on this^23,28,29^ examining the implications of posteriorly acting forces over the thoracolumbar junction as an initiating factor to the development of scoliosis. To show this definitively has challenges, including the serial use of ionising radiation in a longitudinal manner in a large number of growing children without scoliosis. Also, the visual manifestations of a mild scoliosis are often not appreciated until the curve is of a reasonable size, by which the early stages of the development of the scoliosis are in the past and drawing inferences around how the curve developed is not easily possible. Consequently the evidence describing the early stages in the genesis of AIS is lacking.

Dolphens et al.^6^ partially addressed this in 2018 in a cross-sectional study of 1190 children, around the adolescent growth spurt. A logistic regression analysis was used to assess which, of a number of parameters, were related to any coronal asymmetry (defined as the finding of the spinous process lateral to the midline). The paper concluded that sagittal trunk inclination and thoracic kyphosis were associated with an increase in coronal plane trunk asymmetry. The amount of kyphosis (hypokyphosis and hyperkyphosis) was related to the overall shape of the individual, defined as either ‘leaning forwards’ or ‘sway back’ but the further relationship of this finding to the development of AIS was not possible. The methodological issue identified was the cross-sectional nature of the study. It is also of note that the analysis used a logistic regression method, where coronal asymmetry was either present or absent. There was no assessment of the magnitude of the coronal asymmetry in this study nor was the relationship of the coronal asymmetry assessed with regards to the size of the other parameters.

Nissinen et al.^30^ has also assessed the relationships between trunk asymmetry and sagittal profile within the development of scoliosis, noting that trunk asymmetry predicted the future development of scoliosis. Furthermore, Nissinen found that increased kyphosis in females and increased lordosis in males also predicted future AIS. Any observation of the differences in the incidence of AIS related to sex, links to studies that demonstrate a difference in the way that parameters of overall sagittal shape develop during adolescence between males and females^7,31,32^.

This study was designed to re-examine the conclusions of Dolphens et al.^6^ with a truly longitudinal cohort using linear regression. To accomplish this, a surface topography system was used (ISIS2) which provides quantitative information on 3D spinal shape. These 3D parameters include spinal shape, as already described, along with overall sagittal alignment and trunk asymmetry, equivalent to that provided by a scoliometer. As the technique is free of ionising radiation, serial measures are safe. Previous work using ISIS2 has demonstrated its utility as a system for examining back shape on children without visible spinal deformity^1^.

The data presented here reveals the relationships between the parameters that describe the coronal and sagittal shape of the spine as those parameters change. Because it has already been demonstrated that kyphosis and lordosis change with increasing age over the adolescent growth spurt^7,8^, the age of the individuals in this study has been eliminated from the analysis as age is not a co-variate of interest. The analysis shows that the main determinant of coronal asymmetry of the spine is lordosis, with kyphosis and sagittal imbalance playing a lesser role. There are differences seen between males and females and between thoracic and thoracolumbar/lumbar coronal asymmetry. Females are more affected by lordosis, and kyphosis is more important than sagittal imbalance. However, for males, whilst lordosis, and to a lesser degree sagittal imbalance, are again generally the largest coefficients, kyphosis is more important in thoracic coronal asymmetry, whilst sagittal imbalance is more influential in thoracolumbar/lumbar coronal asymmetry. This information is in concordance with our previous work noting the differences in the development of kyphosis and lordosis in the growing child^7,8^. That previous work, combined with this work, suggests that a greater lordosis leads to a greater coronal asymmetry. Of note, kyphosis has a different effect in the thoracolumbar/lumbar coronal asymmetry compared to the thoracic coronal asymmetry as evidenced by the change from a positive to a negative co-efficient. The reasons for this are not clear but this may relate to the overall sagittal shape and a greater kyphosis leading to enhanced stability in intervertebral axial rotation in some fashion, making the development of scoliosis less likely^28,29^.

Consequently, these results both agree and disagree with those of Dolphens et al.^6^ as this paper found an increasing negative sagittal imbalance to be associated with an increasing coronal asymmetry and vice versa. As noted by Dolphens et al.^6^, the effect of the sagittal parameters were different dependant on the anatomical level of the coronal asymmetry seen (thoracic compared to thoracolumbar/lumbar) and this adds to the observation that the two anatomical locations in the spine behave differently.

The results presented here do not definitively link to the Utrecht group, as surface topography does not show individual vertebral bodies and, at present, cannot demonstrate a spinal segment with posteriorly directed forces. However, an understanding of geometry shows that greater lordosis and/or a negative sagittal imbalance will lead to a greater segment of the thoracolumbar spine subjected to posteriorly acting forces. Consequently, this work does provide evidence linking the sagittal shape of the spine to the development of coronal asymmetry and furthers the current understanding of the mechanopathogenesis of AIS. This work shows that the development of coronal asymmetry is related to changes in the parameters that describe sagittal shape. It is known that the parameters of sagittal shape change with age over the adolescent growth spurt and thus a chain of events can be envisioned where that change, seen with increasing age, causes a greater coronal asymmetry in some individuals and the subsequent development of scoliosis. This addition to the biomechanical theory of the genesis of scoliosis would help to explain the development of AIS over the adolescent growth spurt. Whilst the changes in magnitude of the parameters described in this paper are small, the error of the measures described in this paper have established previously^17^ and thus the measures here can be taken as accurate. Furthermore, the findings presented are in agreement with the variability of coronal shape of the spine as seen in the work of Janssen et al.^33^ who showed that there is coronal asymmetry associated with inter-vertebral rotation using an upright MRI scan of individuals without scoliosis.

The strengths of this work are the prospective longitudinal nature of the data. The analysis, with linear mixed effect modelling uses all of the data to create a model for analysis, rather than just a data mean. By breaking the model down into the individual components, the contributions of the different parameters are seen.

The weaknesses of this paper are inherent to a surface topography system which is not a radiograph and so the 3D position of the spine is calculated rather than directly visualised, although the accuracy of this has been demonstrated in the literature^10,11^. Direct validation of the small changes described by the ISIS2 system with other imaging systems in those without scoliosis such as an upright MRI scan (given that radiographs using ionising radiation in those without pathology are not risk free) has not been undertaken. Individual vertebral bodies or the pelvis cannot be seen so the relationship of the spine to the pelvis cannot be commented on. Also, none of the children in the study developed AIS during the data collection, and to the best of our knowledge have not developed AIS since. Consequently, the parameters and coefficients identified, whilst associated with the development of coronal asymmetry, have not been shown to directly lead to the development of AIS.

## Conclusion

In conclusion, the development of coronal asymmetry in a group of children without scoliosis is associated primarily with lordosis but also with sagittal imbalance and kyphosis when in upright stance. These observations add to the evidence that AIS may be driven by relative imbalances in the overall sagittal profile of the spine and the consequences thereof.

## Data Availability

The data that forms the basis of this study is available following reasonable request to the corresponding author.
